# Supplementary material for: On-surface synthesis of planar dendrimers via divergent cross-coupling reaction
Source: Nat Commun. 2019 Jun 3;10:2414. doi: 10.1038/s41467-019-10407-6 (PMC6546735; doi:10.1038/s41467-019-10407-6)
Supplement: Supplementary file 1 — Supplementary Information [file 41467_2019_10407_MOESM1_ESM.pdf]

Supplementary Information for

Li et al, **On-surface synthesis of planar dendrimers via divergent cross-coupling reaction**

On-surface synthesis of planar dendrimers via divergent cross-coupling reaction

Deng-Yuan Li, Shi-Wen Li, Yu-Li Xie, Xin Hua, Yi-Tao Long, An Wang, and Pei-Nian Liu*

Key Laboratory for Advanced Materials, Feringa Nobel Prize Scientist Joint Research Center, State Key Laboratory of Chemical Engineering and School of Chemistry and Molecular Engineering, East China University of Science & Technology, 130 Meilong Road, Shanghai, 200237, China

*Correspondence and requests for materials should be addressed to P.L. (e-mail: liupn@ecust.edu.cn)

Table of contents

Supplementary Note 1. Synthesis of the precursors....S3
Supplementary Note 2. On-surface divergent cross-coupling of one **Br-TPP** with two **ICBP** ..S8
Supplementary Note 3. On-surface synthesis of dendron **2** with four branches....S15
Supplementary Note 4. On-surface synthesis of dendrimer **3** with eight branches S16
Supplementary Note 5. On-surface synthesis of dendrimer **4** with twelve branches....S17
Supplementary References....S18

Supplementary Note 1. Synthesis of the precursors

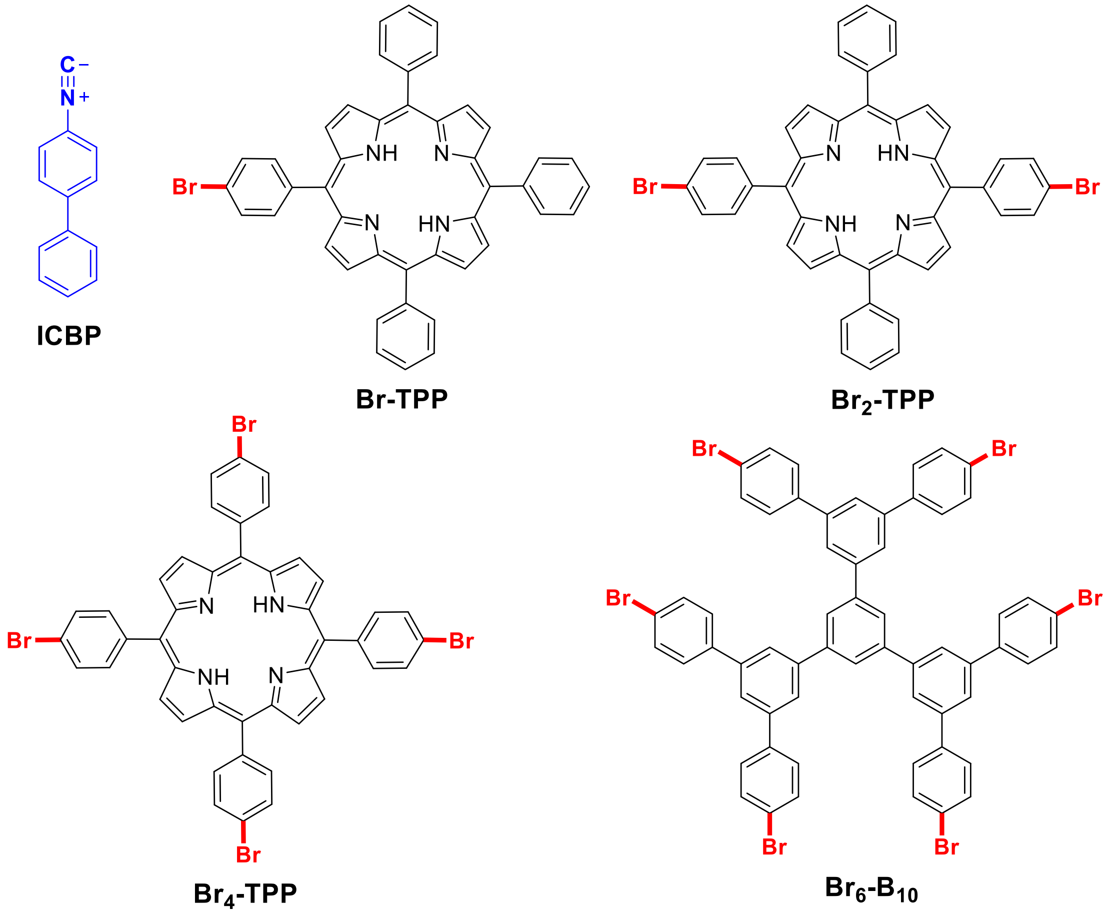

Supplementary Figure 1 | Organic precursors involved in this study.

Precursors **ICBP**,[1,2] **Br-TPP**,[3] **Br$_2$-TPP**,[4] and **Br$_4$-TPP**[5] in Supplementary Figure 1 were prepared according to the methods reported in the literatures. The synthesis of precursor **Br$_6$-B$_{10}$** is as follows:

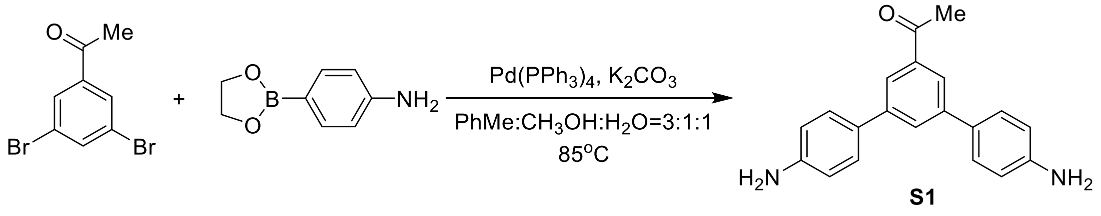

Under argon atmosphere, 1-(3,5-dibromophenyl)ethan-1-one (300 mg, 2 mmol), 4-(4,4,5,5-tetramethyl-1,3,2-dioxaborolan-2-yl)aniline (542 mg，2.4 mmol), Pd(PPh$_3$)$_4$ (115 mg, 0.2 mmol) and K_2CO_3 (2.76 g, 20 mmol) were added in mixed solution of toluene, MeOH and H_2O (volume ratio: toluene : MeOH : H_2O = 3:1:1, 40 mL). The reaction mixture was heated to 85 °C for 12 h. The resulting mixture was added to H_2O and then was extracted with CH_2Cl_2 (3 × 30 mL). The combined organic phases were washed with the saturated aqueous sodium chloride solution (3 ×

30 mL), dried over $MgSO_4$ and the solvent was removed in vacuo. Purification by flash chromatography (CH_2Cl_2/petroleum 3:1) afforded the 1-(4,4''-diamino-[1,1':3',1''-terphenyl]-5'-yl)ethan-1-one (**S1**, 420 mg, 70%) as a pale-yellow solid. **^{1}H NMR (400 MHz, $CDCl_3$, 25 °C):** δ 8.01 (d, J = 1.72 Hz, 2H), 7.88 (t, J = 1.68 Hz, 1H), 7.49 (d, J = 8.52 Hz, 4H), 6.79 (d, J = 8.52 Hz, 4H), 3.79 (br, 4H), 2.68 (s, 3H); **^{13}C NMR (100.6 MHz, $CDCl_3$, 25 °C):** δ 198.66, 146.48, 142.18, 138.16, 130.75, 129.19, 128.31, 124.35, 115.55, 27.05; **HRMS (EI, TOF):** calcd for $C_{20}H_{18}N_2O^+$ $[M]^+$: 302.1419, found: 302.1418.

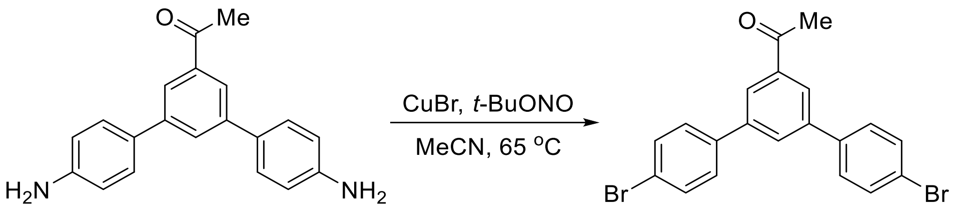

S1 **S2**

Under argon atmosphere, 1-(4,4''-diamino-[1,1':3',1''-terphenyl]-5'-yl)ethan-1-one **S1** (214 mg, 0.5 mmol) was dissolved in MeCN (30 mL) at room temperature. After addition of CuBr 178 mg，1.25 mmol）and *t*-BuONO（129 mg，1.25 mmol, the reaction mixture was stirred at 65 °C for 4 h. The resulting reaction mixture was cooled and then added to HCl-solution (1 M, 10 mL) to quenching the reaction. The mixture was extracted with ethyl acetate (3 × 30 mL). The combined organic phases were washed the saturated aqueous sodium chloride solution (3 × 30 mL), dried over $MgSO_4$ and the solvent was removed in vacuo. Purification by flash chromatography (ethyl acetate/petroleum 10:1) afforded the 1-(4,4''-Dibromo-[1,1':3',1''-terphenyl]-5'-yl)ethan-1-one (**S2**, 150 mg, 70%) as a white solid. **^{1}H NMR (400 MHz, $CDCl_3$, 25 °C):** δ 8.11 (d, J = 1.68 Hz, 2H), 7.90 (t, J = 1.72 Hz, 1H), 7.62 (d, J = 8.56 Hz, 4H), 7.52 (d, J = 8.56 Hz, 4H), 2.70 (s, 3H); **^{13}C NMR (100.6 MHz, $CDCl_3$, 25 °C):** δ 197.83, 141.47, 139.03, 138.53, 132.29, 130.11, 128.99, 126.11, 122.59, 27.03; **HRMS (EI, TOF):** calcd for $C_{20}H_{14}Br_2O^+$ $[M]^+$: 429.9391, found: 429.9391.

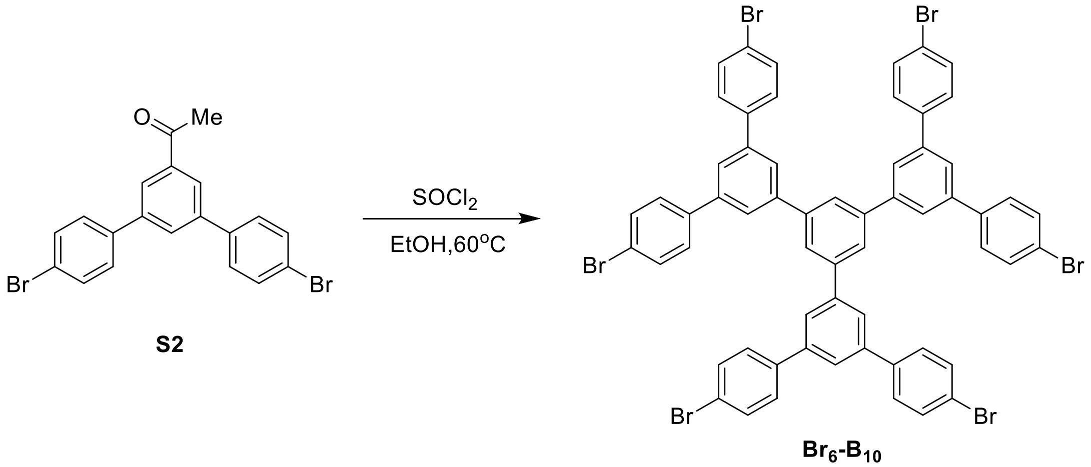

S2

Br_6-B_{10}

Under argon atmosphere, 1-(4,4''-Dibromo-[1,1':3',1''-terphenyl]-5'-yl)ethan-1-one **S2** (150 mg, 0.35 mmol) was dissolved in EtOH (10 mL) at 0 ºC. After addition of $SOCl_2$ (417 mg, 3.5 mmol) and the reaction mixture was stirred at 60 °C for 12 h. The resulting reaction mixture was cooled and then added to H_2O quenching the reaction. The mixture was extracted with ethyl acetate (3 × 30 mL). The combined organic phases were washed the saturated aqueous sodium chloride solution (3 × 30 mL), dried over $MgSO_4$ and the solvent was removed in vacuo. Purification by flash chromatography (CH_2Cl_2/petroleum 25:1) afforded the 4,4''''-Dibromo-5',5'''-bis(4-bromophenyl)-5''-(4,4''-dibromo-[1,1':3',1''-terphenyl]-5'-yl)-1,1':3',1'':3'',1''':3''',1''''-quinquephenyl (**Br_6-B_{10}**, 30 mg, 21%) as a white solid. **Mp:** >300 ºC; **^{1}H NMR (400 MHz, $CDCl_3$, 25 ºC):** δ 7.93 (s, 3H), 7.84 (d, J = 1.52 Hz, 6H), 7.75 (s, 3H), 7.61 (d, J = 8.56 Hz, 12H), 7.56 (d, J = 8.52 Hz, 12H); **^{13}C NMR (100.6 MHz, $CDCl_3$, 25 ºC):** δ 142.65, 142.58, 141.78, 139.75, 132.23, 129.10, 126.16, 125.63, 125.33, 122.30; **HRMS (MALDI-TOF):** calcd for $C_{60}H_{35}Br_6Na^+$ $[M+Na-H]^+$: 1257.7670 , found: 1257.7773.

^{1}H NMR of 4,4''''-Dibromo-5',5'''-bis(4-bromophenyl)-5''-(4,4''-dibromo- [1,1':3',1''-terphenyl]-5'-yl)-1,1':3',1'':3'',1''':3''',1''''-quinquephenyl (Br_6-B_{10})

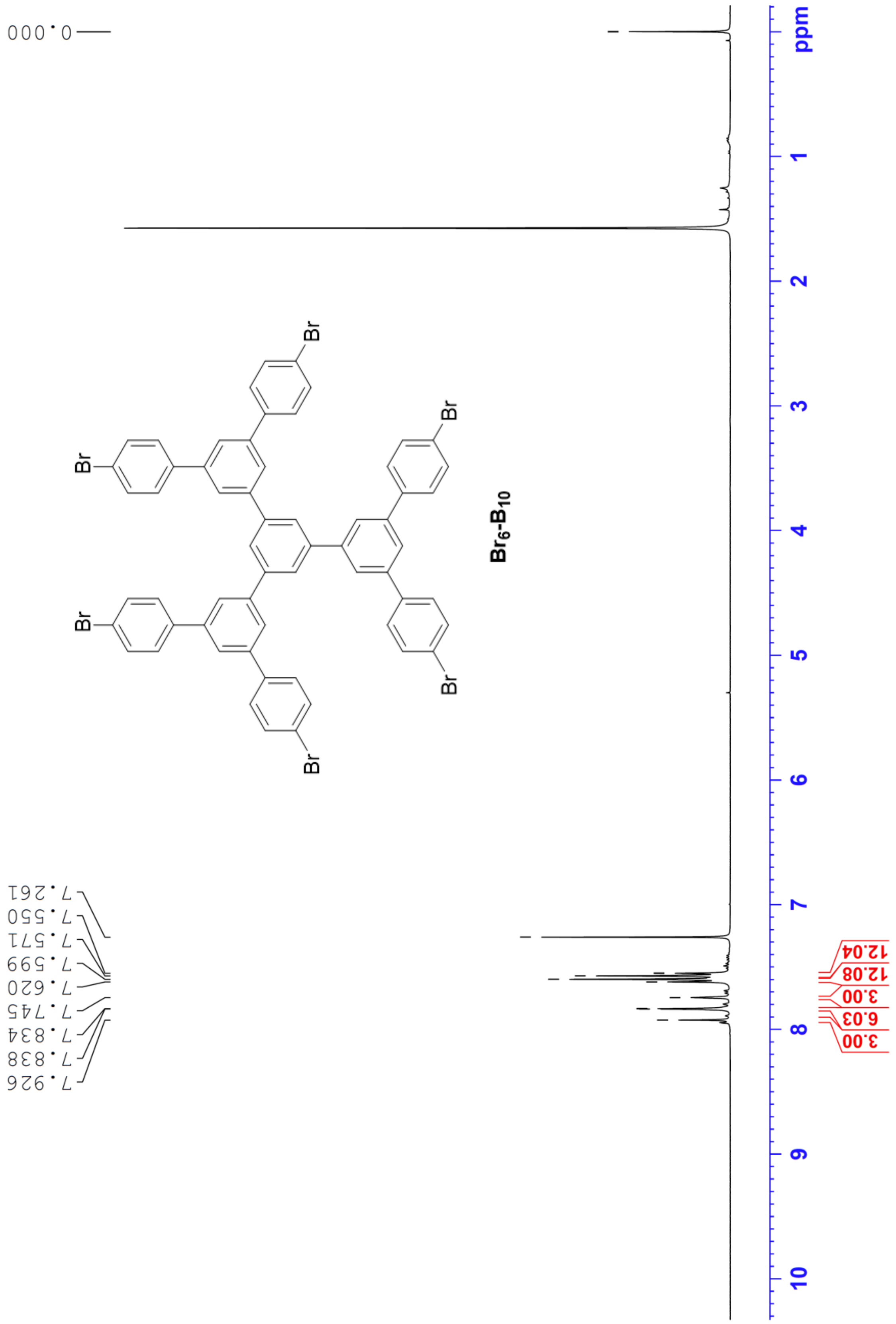

^{13}C NMR of 4,4''''-Dibromo-5',5'''-bis(4-bromophenyl)-5''-(4,4''-dibromo- [1,1':3',1''-terphenyl]-5'-yl)-1,1':3',1'':3'',1''':3''',1''''-quinquephenyl (Br_6-B_{10})

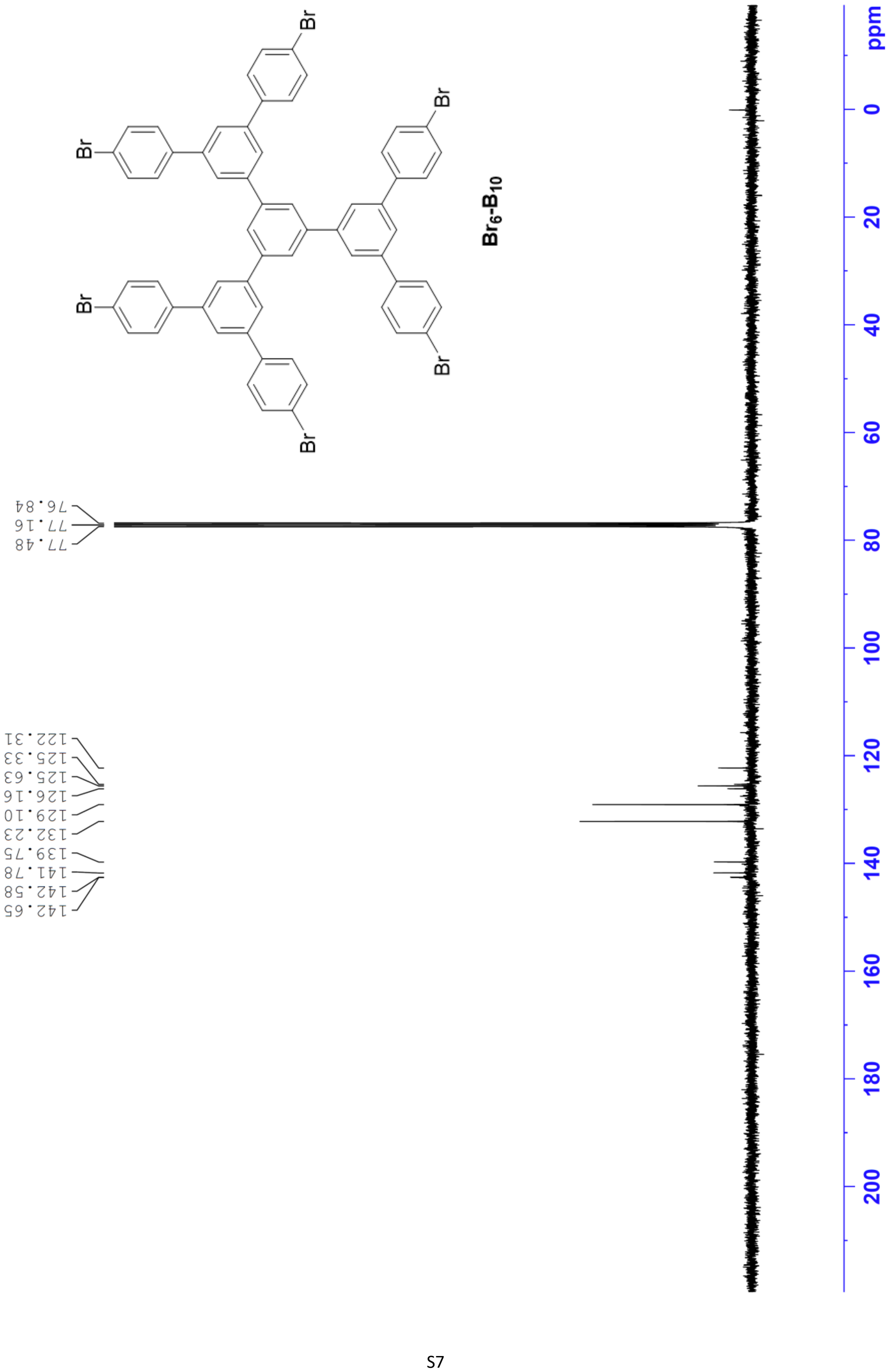

Supplementary Note 2. On-surface divergent cross-coupling of one Br-TPP with two ICBP

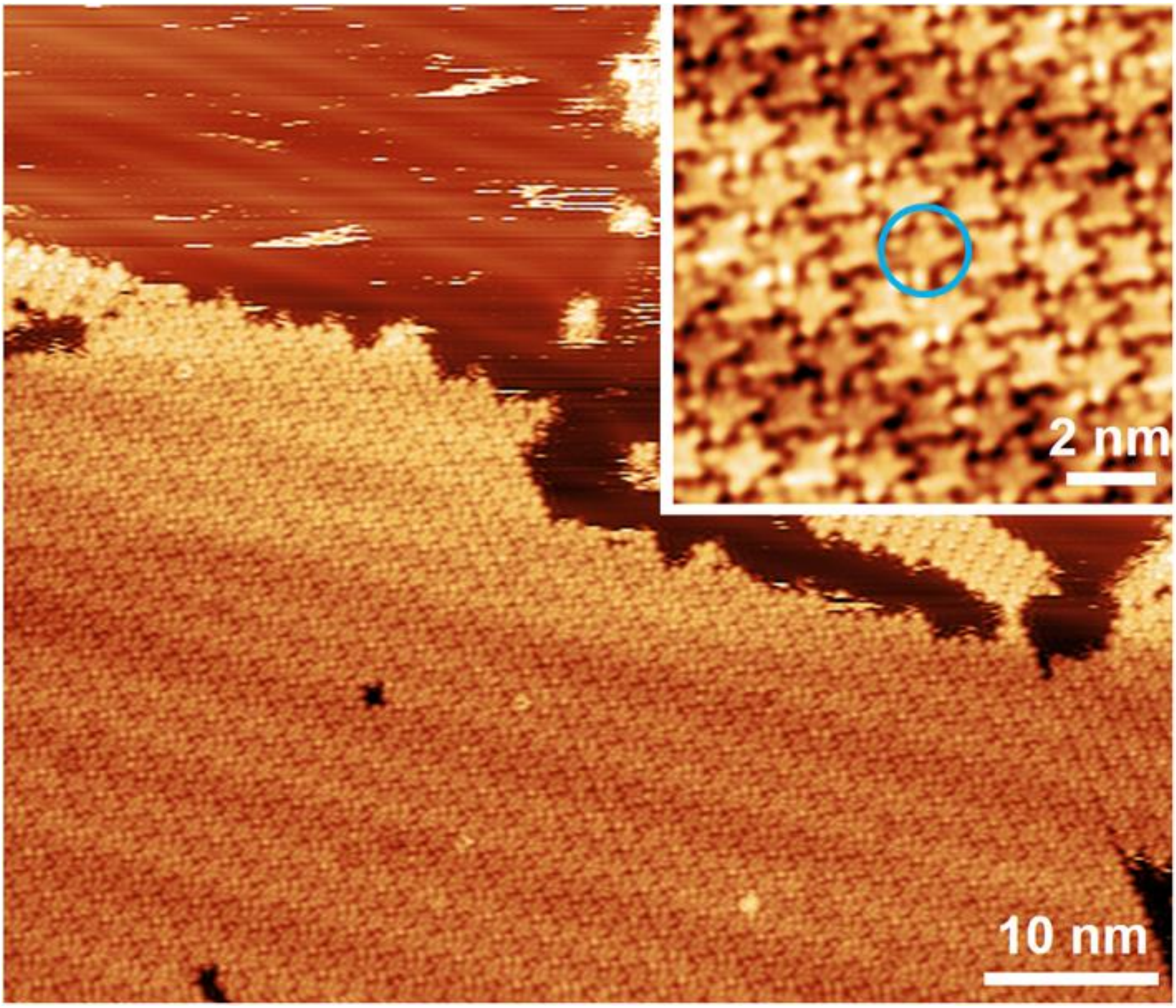

Supplementary Figure 2 | Large-scale STM images for the reaction of **Br-TPP** with **ICBP** on Au(111) after annealing to 403 K for 1 h without Pd deposition. The zoom-in STM image is presented in the inset. The single **Br-TPP** molecule was circled by blue line. Tunneling parameters: *I* = -0.59 nA, *U* = -1.55 V. (inset) *I* = -0.55 nA, *U* = -1.58 V.

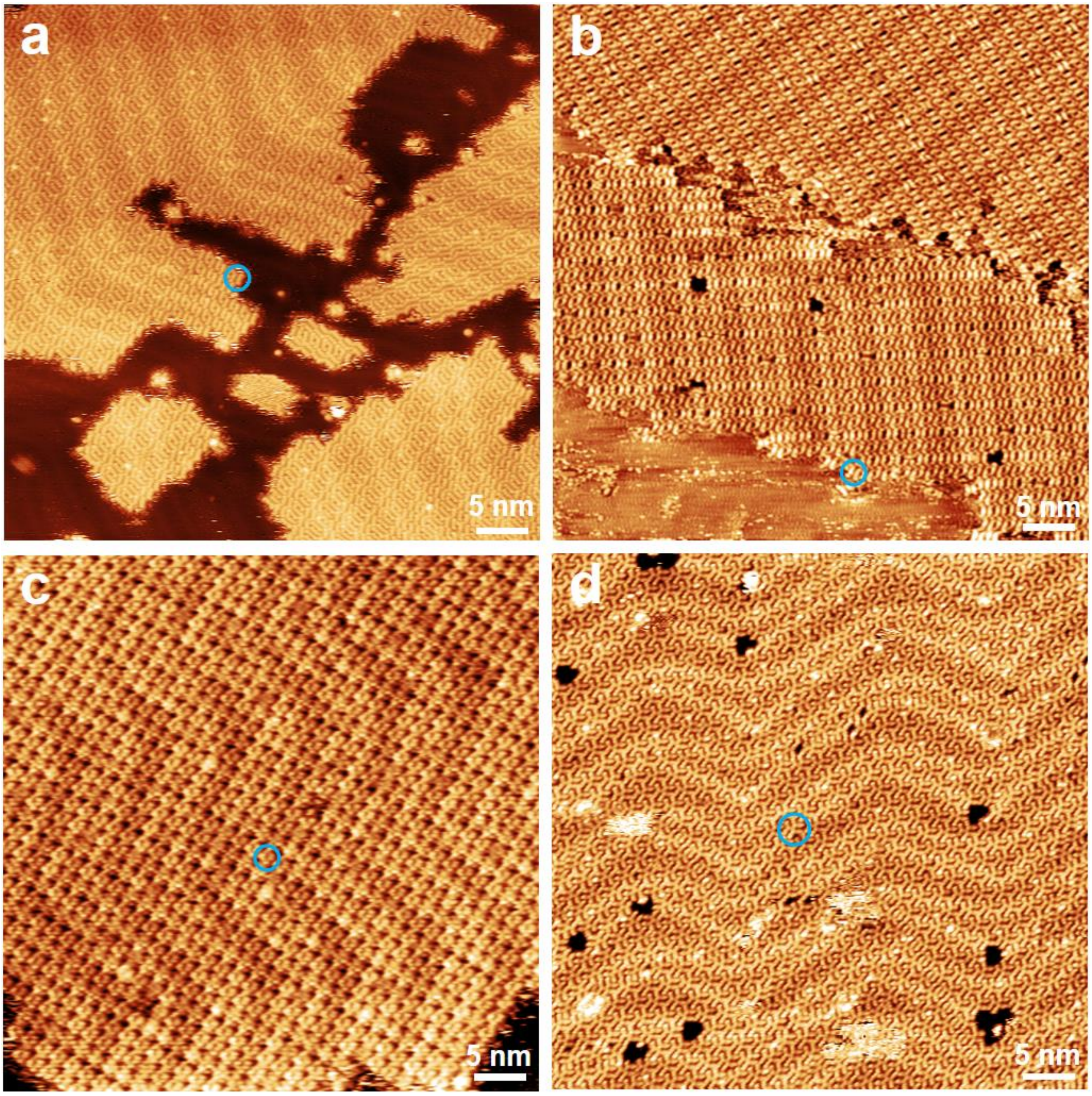

Supplementary Figure 3 | Large-scale STM images for self-assemblies of precursors **Br-TPP**, **Br_2-TPP**, **Br_4-TPP** and **Br_6-B_{10}**. The corresponding single molecule was circled by blue line. Tunneling parameters: (**a**) I = -0.55 nA, U = -1.71 V. (**b**) I = -0.64 nA, U = -1.63 V. (**c**) I = -0.12 nA, U = -1.84 V. (**d**) I = -0.11 nA, U = -1.63 V.

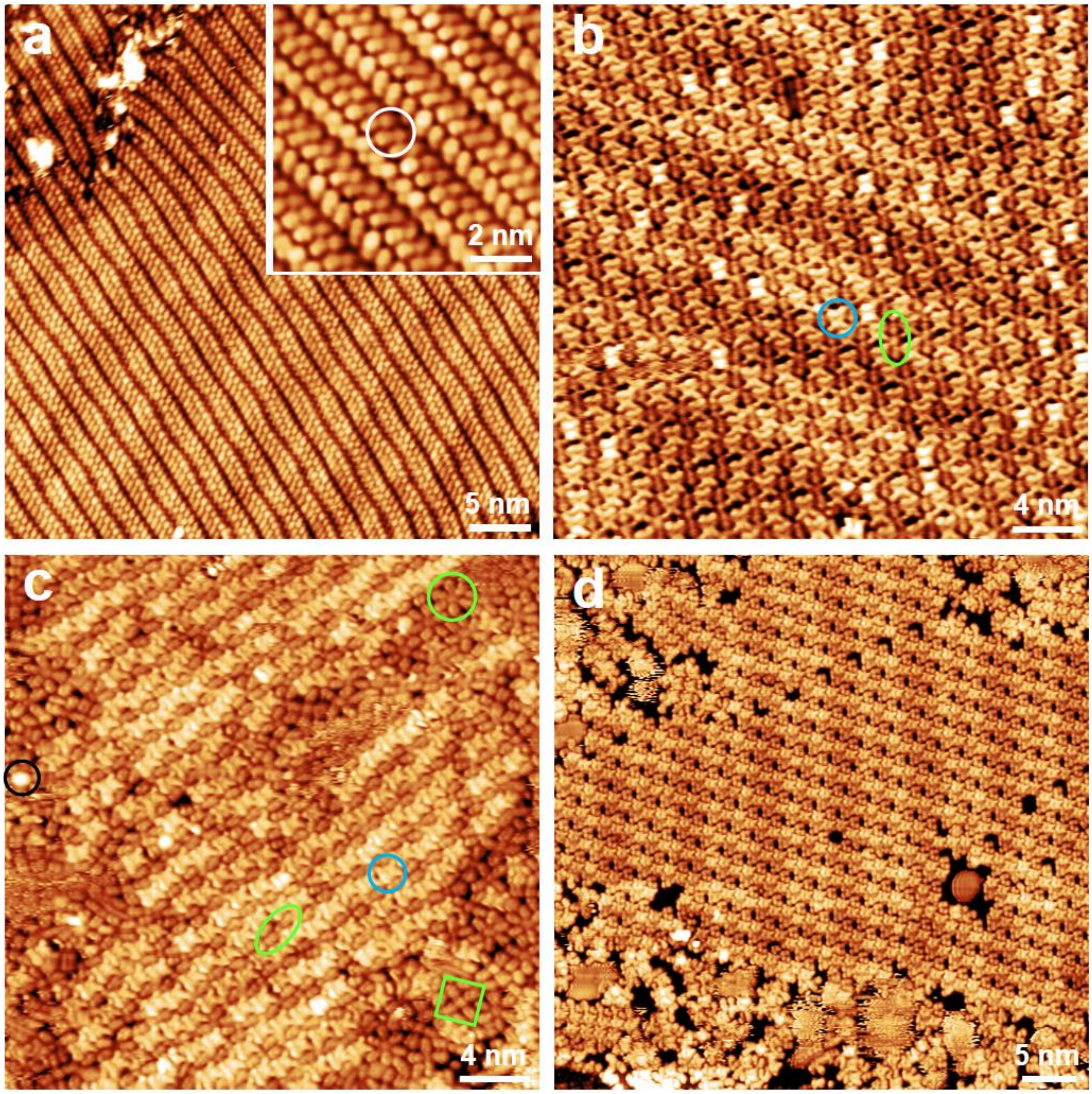

Supplementary Figure 4 | Large-scale STM images for divergent cross-coupling of one **Br-TPP** with two **ICBP** on Au(111). **a**, STM image of self-assembly of **ICBP**. The inset represents a zoom-in STM image of self-assembly of **ICBP**. **b** and **c**, STM images of mixed molecules of **Br-TPP** with **ICBP** before (**b**) and after (**c**) Pd deposition. **d**, STM image for divergent cross-coupling of one **Br-TPP** with two **ICBP** in the presence of Pd after annealing at 403 K for 1 h. The single **ICBP**, **Br-TPP** molecules, and Pd island were circled by white, blue and black lines, respectively. The newly generated coordination structures of **ICBP** with Au and Pd adatoms were labelled by green ellipse, circlet and frame, respectively. Tunneling parameters: (**a**) I = -0.52 nA, U = -1.63 V. (inset) I = -0.07 nA, U = -1.66 V. (**b**) I = 0.07 nA, U = 1.99 V. (**c**) I = -0.13 nA, U = -1.85 V. (**d**) I = -0.13 nA, U = -1.88 V.

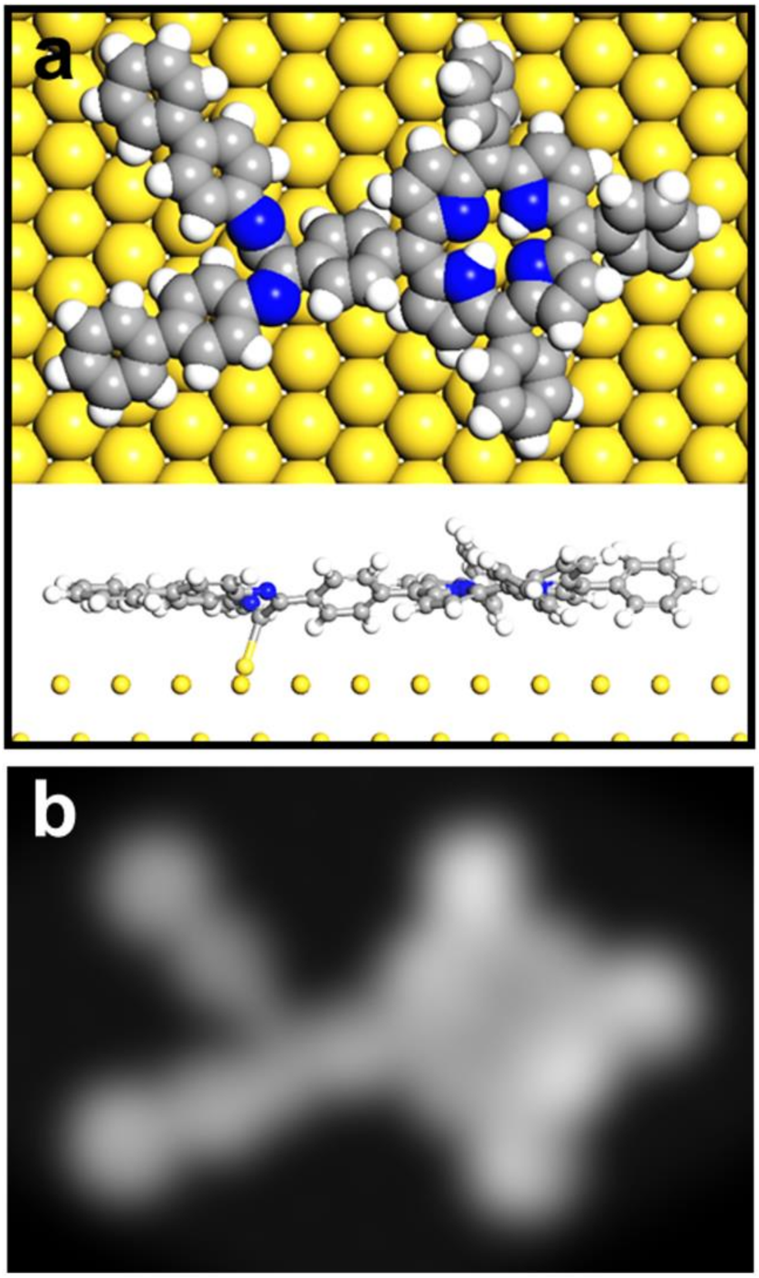

Supplementary Figure 5 | DFT calculated adsorption model (**a**) and simulated STM image (**b**) of **1**.

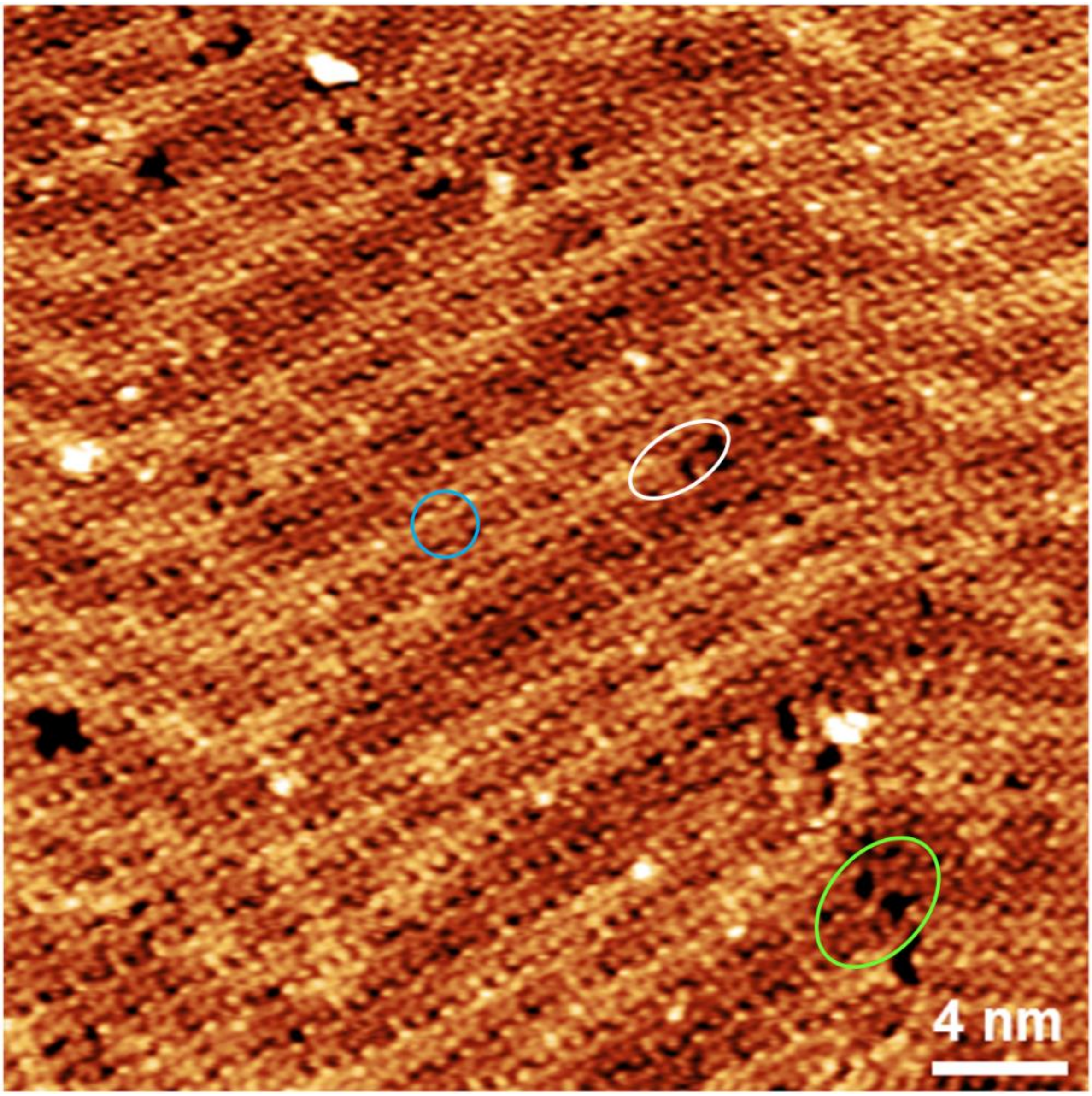

Supplementary Figure 6 | STM image for divergent cross-coupling of one **Br-TPP** with two **ICBP** in the presence of Pd after annealing at 403 K for 1 h (coverage of **Br-TPP**: > 0.8 ML). The single molecules of no coupling, divergent cross-, and homo-coupling were circled by blue, white, and green lines, respectively. Tunneling parameters: I = -0.55 nA, U = -1.16 V.

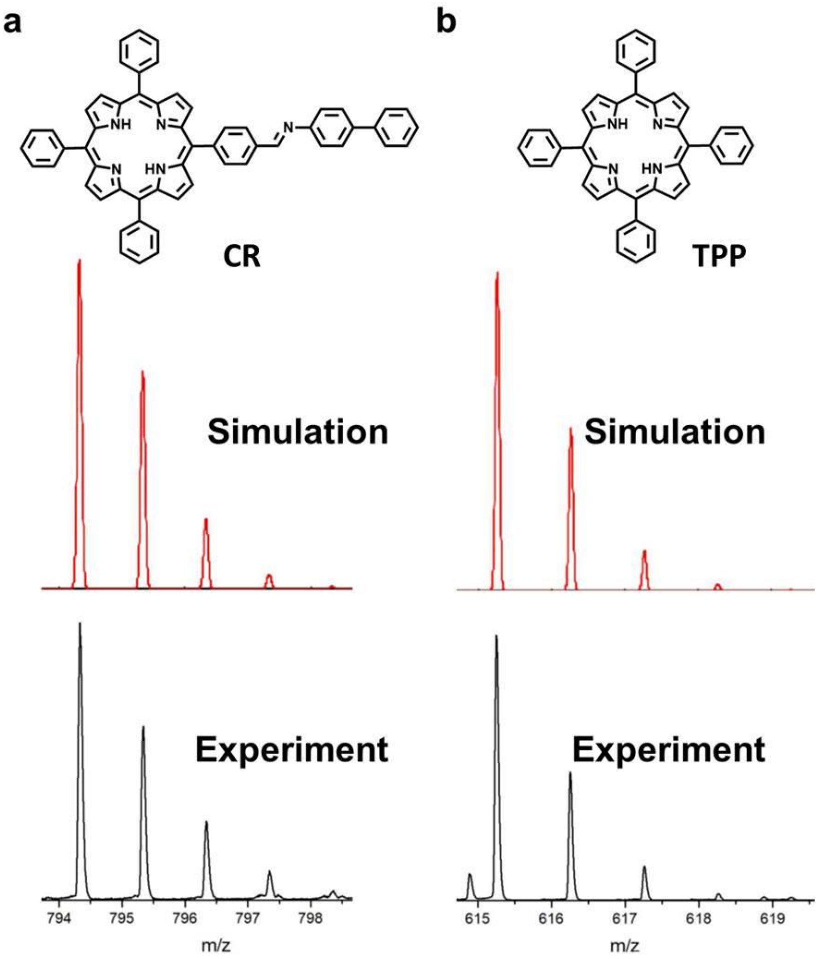

Supplementary Figure 7 | Experimental and simulated isotopic distribution for (**a**) $[M_{CR}+H]^+$ ions of cross-coupling product of one **Br-TPP** with one **ICBP** and (**b**) $[M_{TPP}+H]^+$ ions of debrominated product of **Br-TPP**.

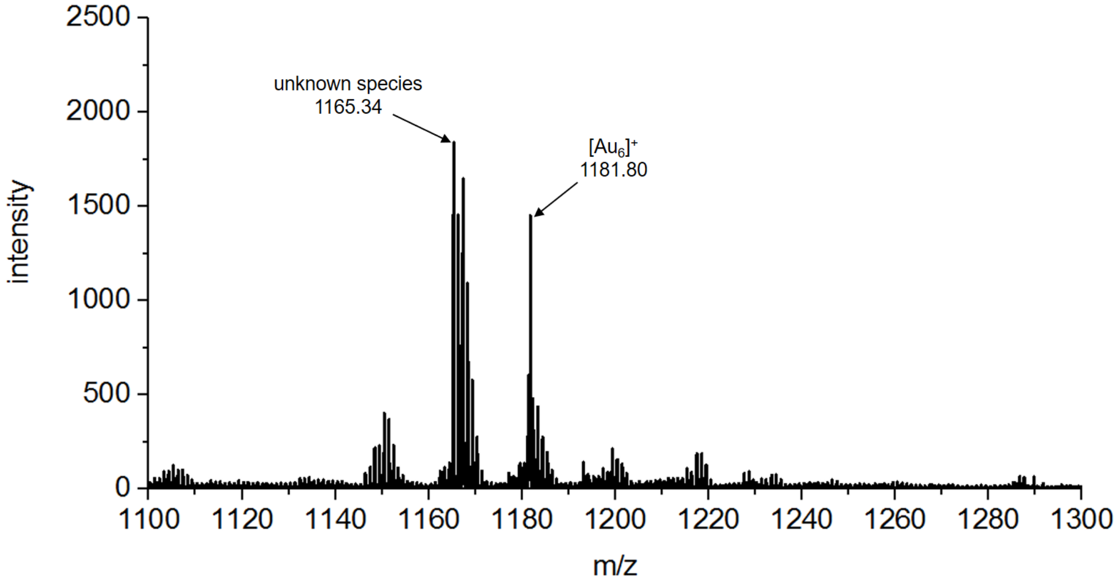

Supplementary Figure 8 | ToF-SIMS spectrum between 1100 and 1300 m/z of **1** on Au(111).

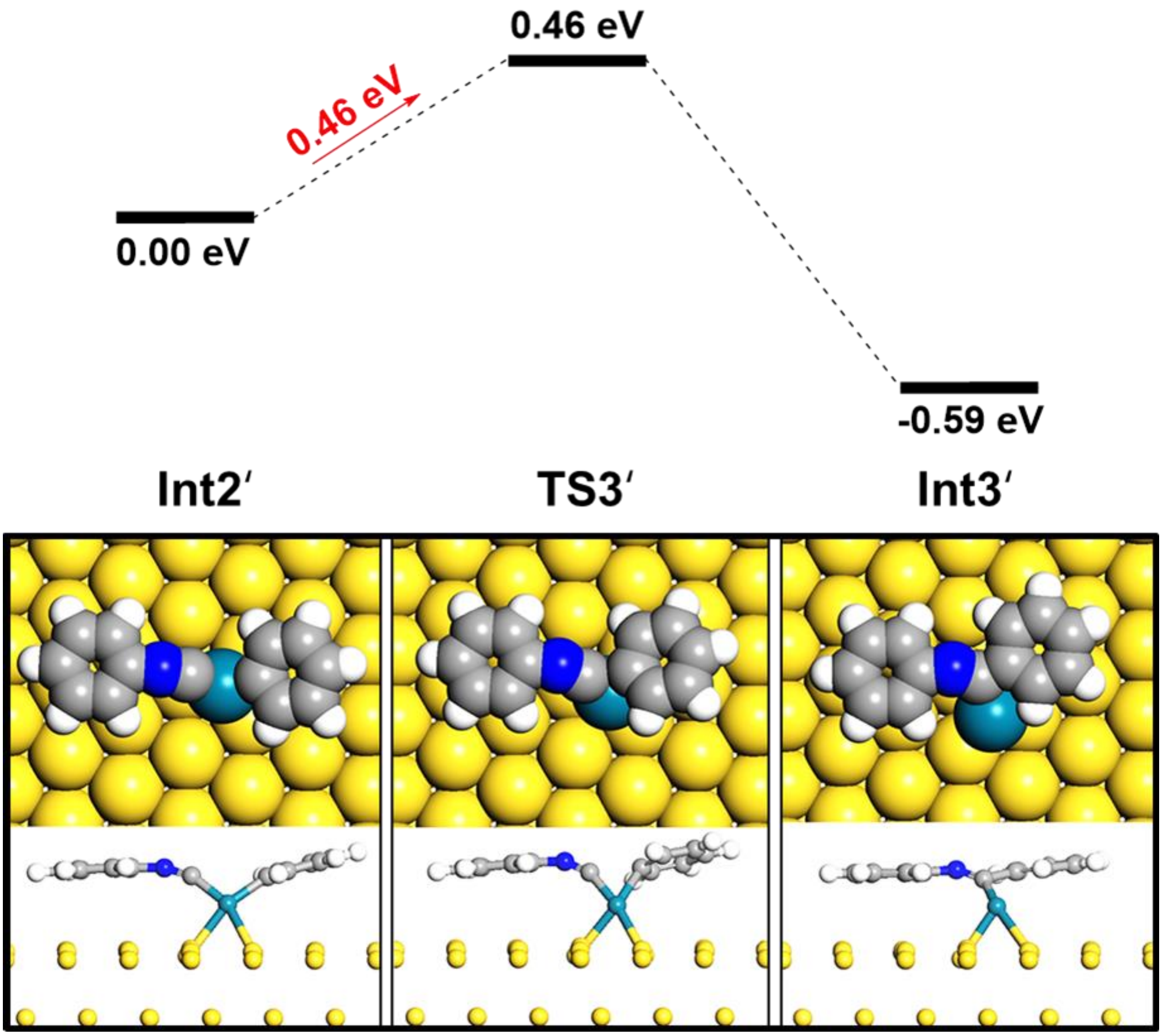

Supplementary Figure 9 | Calculated energy and molecular structures for Pd adatom-promoted addition of benzene radical to isocyanobenzene. The Au(111) surface (6×6) was used in the DFT calculation for Pd adatom-promoted addition of benzene radical to isocyanobenzene.

Supplementary Note 3. On-surface synthesis of dendron 2 with four branches

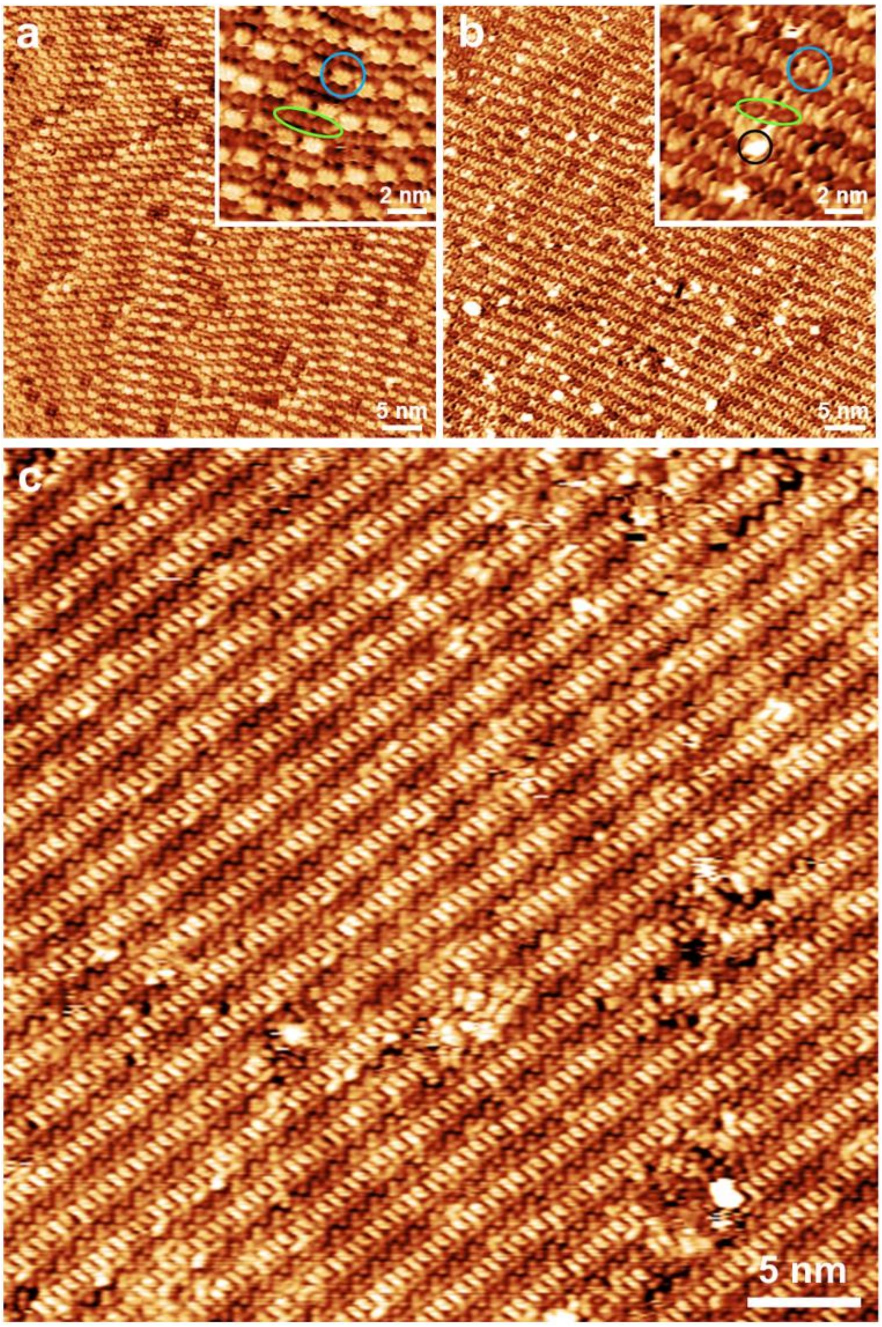

Supplementary Figure 10 | Large-scale STM images for the synthesis of dendron **2** via divergent cross-coupling of one **Br$_2$-TPP** with four **ICBP** on Au(111). **a** and **b**, STM images of mixed molecules of **Br$_2$-TPP** and **ICBP** before (**a**) and after (**b**) Pd deposition. The zoom-in STM images were presented in the corresponding insets. **c**, STM image of self-assembly of dendron **2**. The single **Br$_2$-TPP** molecule and Pd island were circled by blue and black lines, respectively. The newly generated coordination structure of **ICBP** with Au adatoms was labelled by green ellipse. Tunneling parameters: (**a**) I = -0.55 nA, U = -1.63 V. (inset) I = -0.57 nA, U = -1.63 V. (**b**) I = -0.58 nA, U = -1.68 V. (inset) I = -0.60 nA, U = -1.68 V. (**c**) I = -0.10 nA, U = -1.80 V.

Supplementary Note 4. On-surface synthesis of dendrimer 3 with eight branches

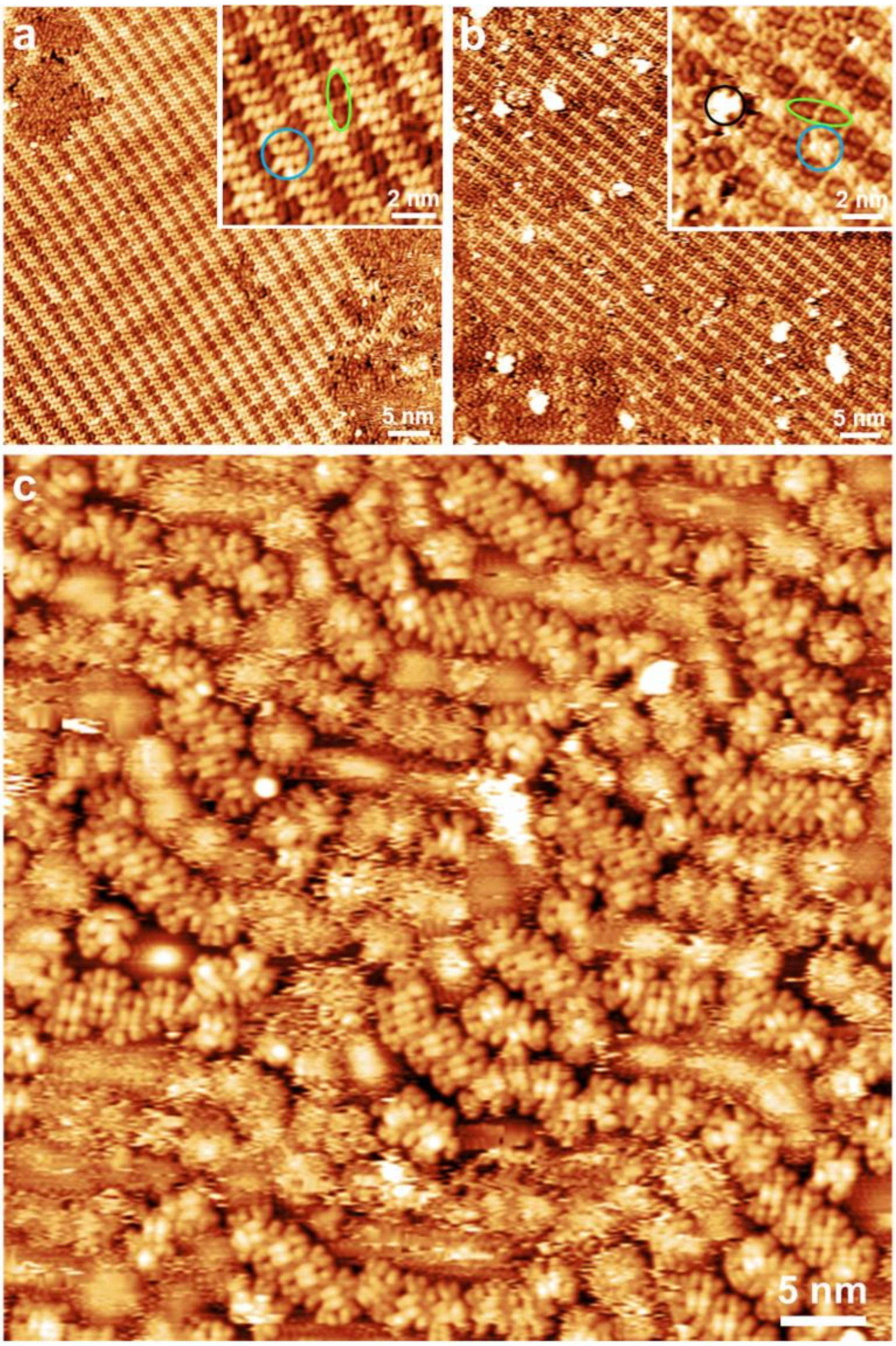

Supplementary Figure 11 | Large-scale STM images for the synthesis of dendrimer **3** via divergent cross-coupling of one **Br_4-TPP** with eight **ICBP** on Au(111). **a** and **b**, STM images of mixed molecules of **Br_4-TPP** and **ICBP** before (**a**) and after (**b**) Pd deposition. The zoom-in STM images were presented in the corresponding insets. **c**, STM image of self-assembly of dendrimer **3**. The single **Br_4-TPP** molecule and Pd island were circled by blue and black lines, respectively. The new generated coordination structure of **ICBP** with Au adatoms was labelled by green ellipse. Tunneling parameters: (**a**) and (inset) I = -0.53 nA, U = -1.68 V. (**b**) I = -0.61 nA, U = -1.63 V. (inset) I = -0.57 nA, U = -1.63 V. (**c**) I = -0.13 nA, U = -1.56 V.

Supplementary Note 5. On-surface synthesis of dendrimer 4 with twelve branches

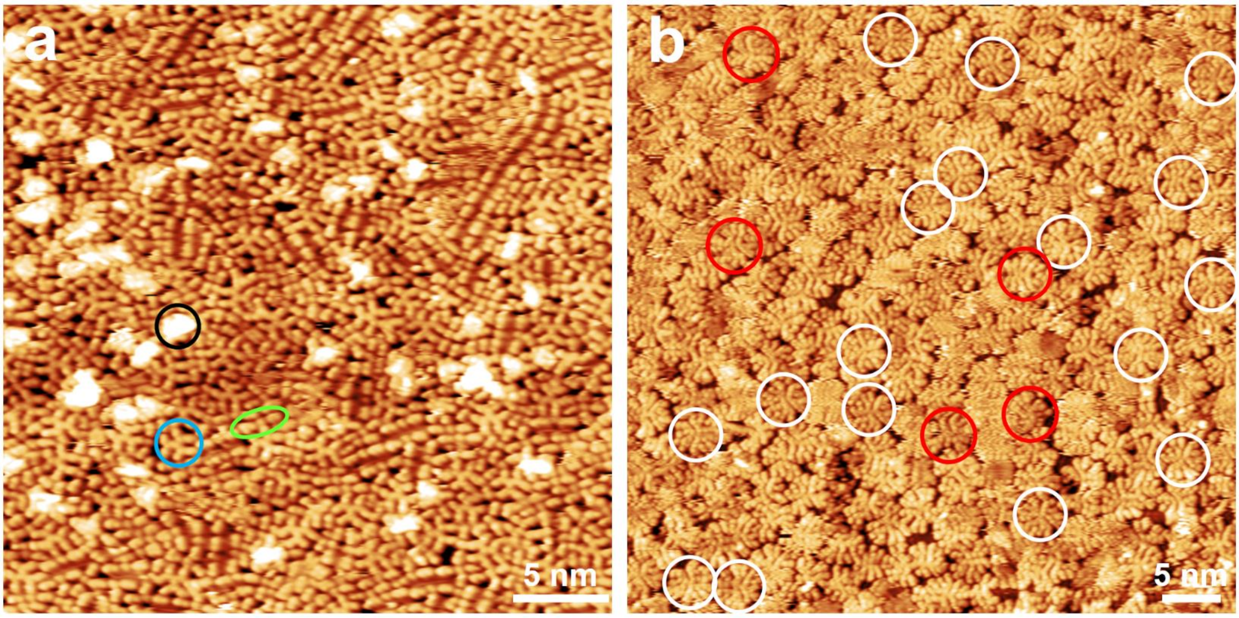

Supplementary Figure 12 | Large-scale STM images for synthesis of dendrimer **4** via divergent cross-coupling of one **Br_6-B_{10}** with twelve **ICBP** on Au(111). **a**, STM image of mixed molecules of **Br_6-B_{10}** and **ICBP** after Pd deposition. **b**, STM image of mixed dendrimer **3** and other partially-branched dendrons. The single **Br_6-B_{10}** molecules and Pd island were circled by blue and black line, respectively. The newly generated coordination structure of **ICBP** with Au adatoms was labelled by green ellipse. The dendrimer **4** and partially-branched dendron were circled by white and red lines, respectively. Tunneling parameters: (**a**) I = -0.56 nA, U = -1.80 V. (**b**) I = -0.71 nA, U = -1.79 V.

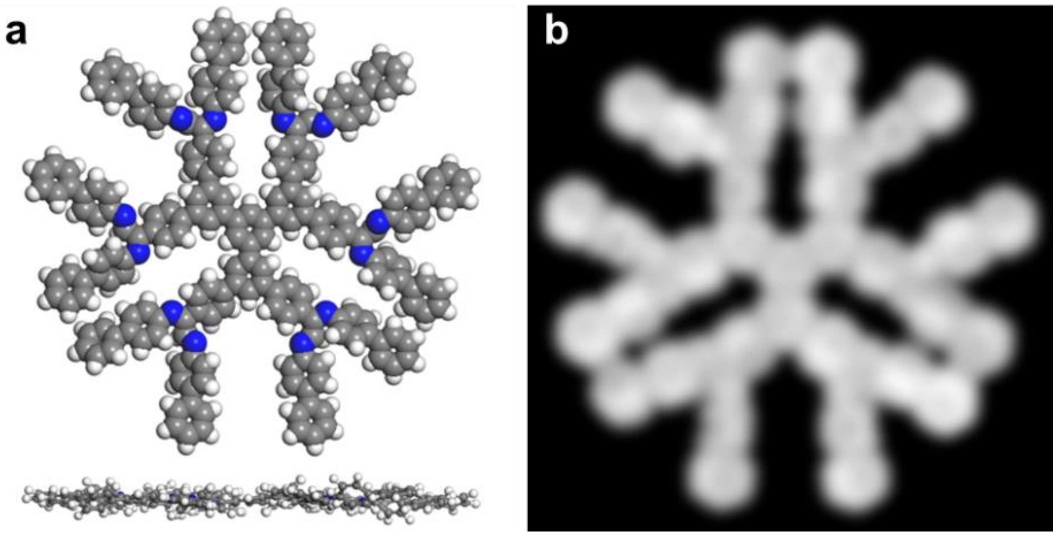

Supplementary Figure 13 | DFT calculated molecule model (**a**) and simulated STM image (**b**) for hydrogenated product of dendrimer **4**.

Supplementary References

1. Dewanji, A., Mück-Lichtenfeld, C., Bergander, K., Daniliuc, C. G. & Studer, A. C−F Activation in Perfluorinated Arenes with Isonitriles under UV-Light Irradiation. *Chem. Eur. J.* **21,** 12295–12298 (2015).
2. Chu, C., Ayres, J. A., Stefanescu, D. M., Walker, B. R., Gorman, C. B. & Parsons, G. N. Enhanced Conduction through Isocyanide Terminal Groups in Alkane and Biphenylene Molecules Measured in Molecule/Nanoparticle/Molecule Junctions. *J. Phys. Chem. C* **111,** 8080–8085 (2007).
3. Li, M., Huang, R., Wu, C., Zuo, H., Lai, G. & Shen, Y. Synthesis and properties of tetrathiafulvalene-porphyrin assemblies. F*ront. Chem. Sci. Eng.* **5,** 422–428 (2011).
4. Lin, T., Shang, X. S., Adisoejoso, J., Liu, P. N. & Lin, N. Steering On-Surface Polymerization with Metal-Directed Template. *J. Am. Chem. Soc.* **135,** 3576–3582 (2013).
5. Wang, X., Wang, H., Yang, Y., He, Y., Zhang, L., Li, Y. & Li, X. Zinc Tetraphenylporphyrin-Fluorene Branched Copolymers: Synthesis and Light-Emitting Properties. *Macromolecules* **43,** 709–715 (2010).
